# The Papilla Stage as a Critical Molecular Transition: *Antp* and Sex-Regulatory Network Orchestrate Cheliped Regeneration in *Eriocheir sinensis*

**DOI:** 10.3390/ani16060982

**Published:** 2026-03-21

**Authors:** Benzhen Li, Yanan Yang, Mengqi Ni, Yourong Liu, Zhaoxia Cui

**Affiliations:** School of Marine Sciences, Ningbo University, Ningbo 315020, China

**Keywords:** *Antp*, *Eriocheir sinensis*, cheliped regeneration, sexual dimorphism

## Abstract

Cheliped regeneration in the *E. sinensis* is a tightly regulated physiological process, yet the molecular regulatory mechanisms underlying sexual dimorphism during regeneration remain unclear. In this study, we combined morphological observation with transcriptomic analysis to systematically investigate the regenerative stage characteristics and sex-related differences. The papilla stage 4 dpa was identified as a pivotal transitional stage, bridging initial wound healing and cellular dedifferentiation (2 dpa) with subsequent redifferentiation and morphogenesis (7 dpa). Morphological sex-based differences characterized by larger regenerating chelipeds in males became prominent by the late stage (28 dpa). Notably, the molecular foundation of sexual dimorphism was found to be established at 4 dpa, significantly preceding the emergence of phenotypic differences. This early divergence was driven by sex-dimorphic endocrine networks: males exhibited preferential expression of genes such as *Fem-1c-like*, *Cyp2L1-like*, *CpAMP1A-like* and *Nedd4-like*, while females showed enrichment in elevated aromatase activity. Weighted gene co-expression network analysis (WGCNA) identified the Hox gene *Antp* as a core hub regulator, exhibiting high co-expression with key epidermal-related genes such as *Cht6*, *Cht2-like* and more. Its suppressed expression at 2 dpa aligned with the requirements for dedifferentiation, whereas its peak at 4 dpa indicated a crucial role in orchestrating appendage patterning and exoskeleton assembly. RNA interference (RNAi) knockdown of *Antp* resulted in obscured differentiation between the propodus and carpus in both sexes and confirmed its regulatory control over downstream targets including *Ubx*, *Bmp2-like*, and *CpAMP1A-like*. This study suggests a putative hierarchical regulatory model in which systemic hormonal signals may integrate *Antp* and other sex-biased regulators to potentially facilitate structured limb regeneration. These findings offer tentative novel insights into the interplay between developmental plasticity and sex-based regulatory divergence in decapod crustaceans.

## Simple Summary

The ability to regenerate chelipeds is vital for the survival of the Chinese mitten crab (*Eriocheir sinensis*), yet the molecular mechanisms driving sexual dimorphism during this process remain unclear. This study combined morphological and transcriptomic analyses to investigate regenerative stages and sex-related differences. We found that while physical differences, such as larger regenerating chelipeds in males, only become prominent at 28 days post autotomy (dpa), the molecular foundation for this divergence is established as early as 4 dpa. This early divergence is driven by sex-specific endocrine networks, with males and females showing distinct gene expression patterns and hormonal activities. Furthermore, we identified the Hox gene *Antennapedia* (*Antp*) as a master regulator. RNAi-mediated knockdown of *Antp* resulted in impaired joint differentiation and confirmed its role in orchestrating appendage patterning and exoskeleton assembly. These findings suggest a putative hierarchical regulatory model, wherein steroid hormones may enter regenerative tissue cells and bind to their corresponding hormone receptors, which could in turn regulate the expression of developmental genes such as *Antp*. Such regulation might potentially modulate downstream transcriptional programs and contribute to the structured regeneration of limbs, providing novel speculative insights into crustacean developmental biology.

## 1. Introduction

Regeneration, the spellbinding biological feat of mending damaged bodies by restoring functional tissues and complex organs, stands as one of the most enthralling puzzles in developmental biology [1,2]. The core of this process hinges on a precise cellular transition: mature cells at the wound site first undergo dedifferentiation, through which they shed their specialized phenotypic traits and undergo reprogramming to revert to a progenitor cell state [3,4]. These multipotent cells subsequently aggregate to form a proliferative blastema, and then proceed to undergo redifferentiation to faithfully recapitulate the missing tissue structure [5]. While the core stages from dedifferentiation to redifferentiation are conserved across a broad range of taxa, the molecular switches governing these transitions, particularly regarding their interaction with biological sex, have yet to be fully elucidated.

Crustaceans represent excellent models for studying the mechanisms of appendage regeneration [6]. A notable example is the Chinese mitten crab, *E. sinensis*, which is an economically significant aquaculture species widely distributed across East Asia [7]. In 2024, the production of Chinese mitten crab in China reached 894,395 tonnes, accounting for 75.75% of the total crustacean aquaculture output in the country [8]. In this species, the chelipeds (pincer-like appendages) are indispensable not only for foraging and defense, but also as important secondary sexual characteristics. Male individuals begin to gradually develop larger claws and denser setae during the juvenile crab stage [9,10]. Both in natural habitats and intensive aquaculture systems, *E. sinensis* frequently undergoes cheliped autotomy resulting from intraspecific agonistic interactions. Such impairments substantially compromise the organism’s survival, growth performance, and overall economic value [11,12].

The regenerative cycle of the cheliped encompasses multiple sequential physiological phases, among which three key stages are recognized as core drivers of the regenerative process: the scab stage, papilla stage, and limb bud stage [13,14]. Notably, the papilla stage serves as a core molecular regulatory node, marking the transition from wound healing to morphogenesis [15]. While substantial foundational research has been conducted on limb regeneration in crustaceans, and core signaling pathways including Wnt, Notch, and TGF-β have been well-documented as key regulators in this process, studies focusing on potential sexual dimorphism during the early phase from cellular reprogramming to redifferentiation remain scarce [16,17,18]. In particular, the sex-differential activation patterns and regulatory effects of these pathways in male and female individuals have not yet been thoroughly elucidated. Given that the chelipeds of male *E. sinensis* are significantly more robust and morphologically complex than those of females, investigating how sex-differential regulatory networks modulate the regenerative process is of great importance.

The genetic identity and spatial patterning of segments are governed by the highly conserved Hox gene family [19]. Among these, *Antp* acts as a master regulator of appendage identity and growth in crustacean [20]. *Sex combs reduced* (*Scr*), another critical Hox gene, is involved in the regulation of appendage patterning in *Drosophila* and plays a central role in the development of sex combs, a structure associated with sexual dimorphism [21]. As a member of the Hox gene family, *Antp* may similarly function in the development of chelipeds, which represent secondary sexual characteristics, in *E. sinensis*. However, the functional requirement of *Antp* during the early stages of cheliped regeneration in *E. sinensis*, as well as its potential synergy with sex differentiation-related genes, has yet to be characterized.

In this study, comparative transcriptomic profiling was employed to systematically analyze the regenerative progression across three pivotal stages in both male and female *E. sinensis*. Our results identify the papilla stage as the primary window for sex-biased gene expression, featuring key candidate regulators such as *Antp*, *Cyp2L1-like*, *CpAMP1A-like*, *Fem-1c-like*, *Ftz-f1-like*, *E75*, and *Nedd4-like*. Functional validation via RNAi further demonstrated that *Antp* is indispensable for the transition from the papilla stage to the growth stage; silencing of *Antp* not only significantly retards the regeneration rate but also downregulates the expression of *Ubx*, *Bmp2-like* and *CpAMP1A-like*. These findings provide novel insights into how Hox genes and sex-biased pathways coordinately govern the intricate transition from cellular dedifferentiation to redifferentiation in sexually dimorphic organs.

## 2. Materials and Methods

### 2.1. Tissue Sampling and RNA Isolation

The juvenile Chinese mitten crabs used in this experiment were obtained from Nanjing, Jiangsu Province, with an average body weight of 15 ± 3.8 g. The crabs were housed in 6 rectangular tanks (1 m × 0.6 m × 0.4 m) for a 3 day acclimation period, under conditions of a constant water temperature of 20 ± 1 °C, natural photoperiod, and dissolved oxygen content above 5 mg/L. During acclimation, the crabs were fed a mixed diet of corn, soybean meal, and manila clam (*Ruditapes philippinarum*) at an approximate ratio of 1:1:1, with a daily feeding allowance of roughly 5% of their body weight and daily water renewal. After acclimation, 100 males and 100 females in the intermolt stage with intact appendages were selected, anesthetized on ice, and right cheliped autotomy was induced by manual pressure. After no hemolymph exuded from the wound, the crabs were stocked at 40 individuals per tank. To simulate the real aquaculture environment, each tank was co-cultured with 20 males and 20 females. Morphological changes were recorded by photography after autotomy. Intact regenerating cheliped tissues were collected separately by sex at 2, 4, 7 and 28 dpa, with 6 individuals sampled for each sex at each stage, and the regenerating tissue from a single crab served as one biological replicate.

Total RNA was isolated from regenerating cheliped tissues using RNAiso Plus (TaKaRa, Shiga, Japan) per the manufacturer’s protocol. RNA quality and concentration were quantified with a NanoDrop™ One C Spectrophotometer (Thermo Fisher Scientific, Waltham, MA, USA). For samples at 2, 4 and 7 dpa, three biological replicates were prepared for both sexes and submitted to LC-Bio Technologies (Hangzhou, China) for transcriptome sequencing, designated as M/F2dpa1-3, M/F4dpa1-3, and M/F7dpa1-3.

### 2.2. Transcriptome Sequencing and Data Processing

Paired-end sequencing (PE150, 2 × 150 bp read length) was conducted on the Illumina NovaseqTM 6000 platform (Illumina, San Diego, CA, USA). Raw sequencing reads were first subjected to quality filtering using Trimmomatic-0.39 to generate clean data [22], followed by quality assessment of the filtered reads with FastQC-v0.11.5. The qualified clean data were then aligned to the reference genome using Hisat2-v2.2.1 [23,24]. The resulting SAM alignment files were converted to binary BAM format via Samtools-1.2.0 [25]. Finally, Stringtie-v2.2.3 was employed to quantify gene expression levels [26], with Transcripts Per Kilobase of exon model per Million mapped reads (TPM) values calculated accordingly [27].

### 2.3. Transcriptome Data Analysis

DEG analysis was performed on the raw count gene expression matrix using the DESeq2-3.22 package [28]. DEGs were screened with the thresholds of |log2 fold change (log2FC) > 1 and false discovery rate (FDR) < 0.05. Volcano plots visualizing DEGs were constructed using the ggplot2 package [29]. Subsequently, functional annotation of DEGs was conducted via sequence alignment against the non-redundant database (https://ftp.ncbi.nlm.nih.gov/blast/db/FASTA/nr.gz, accessed on 27 April 2025) using Diamond-2.1.8 and Blast-2.14.0 [30,31]. For functional classification and pathway enrichment, DEGs were annotated based on the GO (http://geneontology.org, accessed on 12 May 2025) and Kyoto Encyclopedia of Genes and Genomes (KEGG, https://www.kegg.jp, accessed on 12 May 2025). GO, KEGG, and Gene Set Enrichment Analysis (GSEA) were performed using the OmicStudio tools (https://www.omicstudio.cn/tool), with all expressed genes serving as the background gene set. For GSEA, the entire gene expression matrix was used to identify coordinated biological changes and significant enrichment patterns. In addition, some figures were generated using BioGDB (https://biogdp.com) [32].

### 2.4. Cluster Analysis of Gene Expression Patterns and Validation by qRT-PCR

Cluster analysis and visualization of gene expression patterns across transcriptome data from different time points were conducted via the Mfuzz 2.54.0 package [33]. Venn diagrams were constructed to visualize the overlap of DEGs among different time points using the VennDiagram v1.7.4 package [34]. Primers were designed using SnapGene 6.02 (https://www.snapgene.com) (Table S1). *β-actin* served as the reference gene for normalization. Following total RNA extraction, reverse transcription was performed, and quantitative reverse transcription polymerase chain reaction (qRT-PCR) was carried out using the PerfectStart^®^ Green qPCR SuperMix (TransGen Biotech, Beijing, China). Each experiment group included 4 biological replicates and 3 technical replicates to ensure reliability. Relative gene expression levels were computed using the 2^−ΔΔCt^ method [35]. All data were presented as the mean ± standard deviation (SD). Normality of the data distribution was assessed using the Shapiro–Wilk test via GraphPad Prism 10.1.12. Homogeneity of variances was examined by Levene’s test with SPSS 17.0 software [36]. According to the characteristics of the gene-related data, subsequent statistical analysis was performed using mixed-model analysis of variance (ANOVA) with GraphPad Prism. Bar graphs were constructed using GraphPad Prism 10.1.12.

### 2.5. Weighted Gene Co-Expression Network Analysis

WGCNA was constructed using the WGCNA Shiny v0.0.6.230118 embedded in TBtools-II v2.360 [37,38]. Prior to network establishment, TPM values were subjected to logarithmic transformation for data normalization. To guarantee high-quality data input, genes with expression levels below 1 in over 50% of the samples were filtered out. The key parameters for network construction were set as follows: soft threshold was fixed at 10, module tree height was set to 0.25, and the minimum number of genes per module was defined as 30. The cytohubba v0.1 plugin was used to screen hub genes of the modules and construct the corresponding network graphs (https://apps.cytoscape.org/apps/cytohubba, accessed on 21 May 2025).

### 2.6. RNA Interference

Primers for dsRNA synthesis of *Antp* and *eGFP* were designed (Table 1). dsRNA was synthesized via in vitro transcription using a dsRNA Synthesis Kit (Sangon Biotech, Shanghai, China). The dsRNA was dissolved in sterile, enzyme-free PBS, with a small amount of phenol red added as an indicator. To avoid degradation, the mixture of dsRNA and phenol red was freshly prepared immediately before each injection.

After cheliped autotomy, crabs were monitored for scab formation to ensure all individuals entered the scab stage; those that failed to form scabs were excluded before injection. At 3 dpa, dsRNA was injected into the coxopodite of the regenerating cheliped at a dose of 3 μg per gram of crab body weight.

First, RNAi efficiency was verified. Then, according to the effect of injection frequency on survival rate, an injection interval of 72 h was determined, and a total of three rounds of injections were performed. The dsRNA-*Antp* group included 30 males and 30 females, while the dsRNA-*eGFP* control group included 25 males and 25 females.

## 3. Results

### 3.1. Morphological Observation and Sequencing Data of Cheliped Regeneration of E. sinensis

**Figure 1:**
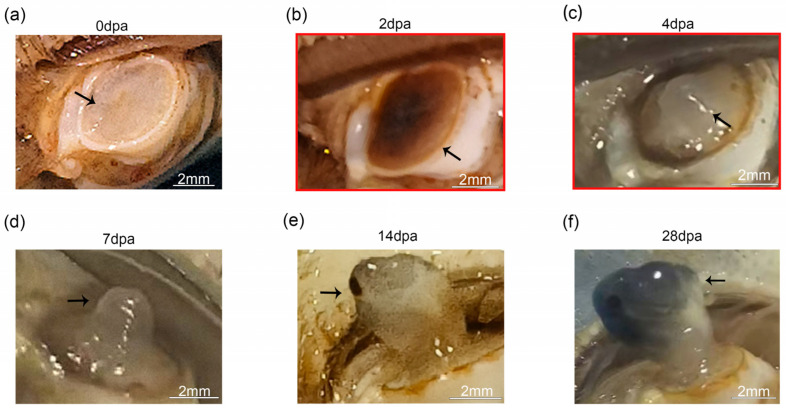
Morphological observation on cheliped regeneration of *E. sinensis*. (**a**) The cheliped immediately after autotomy. (**b**) Regeneration status at 2 dpa, where a wound scab formed. (**c**) Regeneration status at 4 dpa; the wound scab shed, and a papilla emerged. (**d**) Regeneration status at 7 dpa, characterized by the outgrowth of a regenerative limb bud. (**e**) Regeneration status at 14 dpa, by which time a nearly complete and transparent cheliped had formed. (**f**) Regeneration status at 28 dpa, during which body surface melanization and segmental swelling occurred. The stages marked by red boxes were selected for transcriptome sequencing. The black arrows indicate the autotomy wound and regenerated tissue.

The majority of *E. sinensis* individuals regenerated a fully functional cheliped after a single molting event post autotomy. Within hours post amputation, the wound site appeared transparent, accompanied by a minor exudation of hemolymph. Subsequently, black punctate structures emerged at the wound surface (Figure 1a). By 2 dpa, a black scab had completely covered the amputation site (Figure 1b). Around 4 dpa, this scab began to detach, revealing a transparent, papillary structure enclosed by a thin, translucent membrane (Figure 1c). By 7 dpa, a prominent regenerative limb bud became visible (Figure 1d). By approximately 14 dpa, a structurally intact but transparent new cheliped had formed (Figure 1e). During the following two weeks, the segments of the nascent cheliped became fully encased in a black membrane and exhibited significant swelling (Figure 1f).

Transcriptome sequencing was performed on the regenerating chelipeds of *E. sinensis* at 2, 4, and 7 dpa based on the morphological observation. Following quality filtering, a total of 129.38 GB of high-quality data were generated for subsequent bioinformatic analysis. The average proportion of Q30 bases (corresponding to a sequencing error rate of <0.1%) reached 95.76%, with an average GC content of 49.63% and an average mapping rate of 78.62% (Table S2).

### 3.2. Molecular Transition from Wound Healing to Blastema Formation During Early Limb Regeneration in E. sinensis

**Figure 2:**
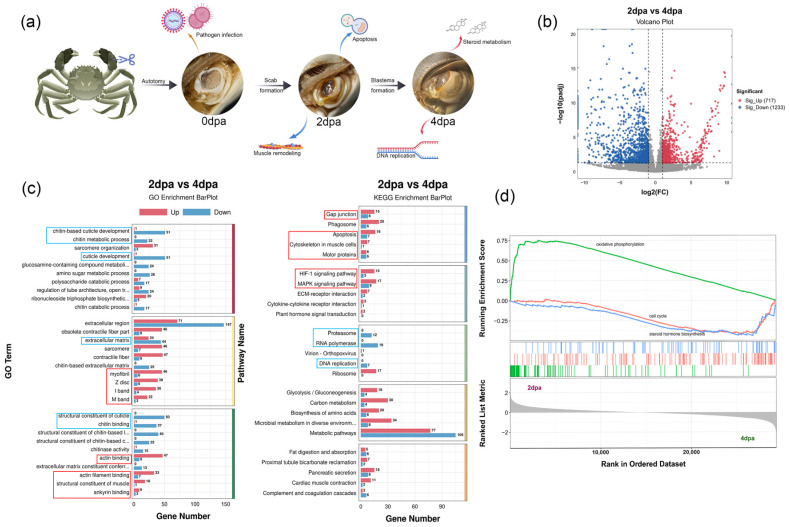
Molecular characteristics of regenerative wound healing and blastema formation. (**a**) Early stages of cheliped regeneration in *E. sinensis* and associated cellular and molecular events. (**b**) Volcano plot of DEGs between 2 dpa and 4 dpa stages: red indicated up-regulated genes at 2 dpa, and blue indicated up-regulated genes at 4 dpa. (**c**) GO and KEGG enrichment analysis of DEGs between 2 dpa and 4 dpa stages: red boxes represented terms or pathways enriched at 2 dpa, and blue boxes represented those enriched at 4 dpa. (**d**) GSEA results of 2 dpa and 4 dpa stages. Transcriptome data from males and females were combined for this comparison; only differences among developmental stages were analyzed, *n* = 6.

Following cheliped autotomy, *E. sinensis* underwent a sequence of wound healing and blastema formation, marking a pivotal transition from wound repair to the initiation of regeneration. The 2 dpa stage represented the scab phase of healing, whereas by 4 dpa, the formation of a papilla was observed, which set the stage for subsequent morphological patterning (Figure 2a). Transcriptomic profiling identified 1950 DEGs at 2 dpa compared to 4 dpa, comprising 717 up-regulated and 1233 down-regulated genes (Figure 2b).

At 2 dpa, the molecular signature was defined by tissue remodeling and acute stress responses. DEGs were significantly associated with myofibril assembly and cytoskeletal dynamics involving actin and ankyrin binding activities (Figure 2c). The activation of HIF-1 and MAPK pathways, coupled with high oxidative phosphorylation in GSEA, suggested intense cellular homeostasis and oxidative stress during initial wound closure (Figure 2c,d).

By 4 dpa, the transcriptional landscape shifted toward structural reconstruction and rapid cell proliferation. This stage was characterized by enriched chitin metabolic processes and cuticle development, signaling the onset of new tissue formation (Figure 2c). The activation of DNA replication and cell cycle-related pathways reflected the high proliferative capacity of nascent blastema cells. Notably, the upregulated steroid metabolism observed in GSEA indicated that the initiation of regeneration might be regulated by steroid hormones (Figure 2d).

### 3.3. Molecular Transition from Blastema Development to Morphogenesis in the Regenerating Cheliped of E. sinensis

**Figure 3:**
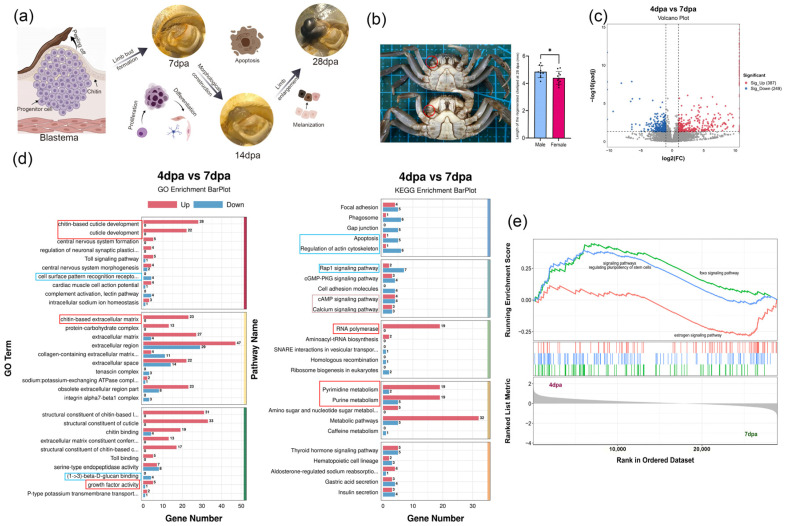
Molecular characteristics of regenerative redifferentiation in *E. sinensis* chelipeds. (**a**) Cellular and molecular events associated with redifferentiation and morphological patterning during cheliped regeneration in *E. sinensis*. (**b**) Morphological differences in regenerated chelipeds between males and females at 28 dpa; the regenerated chelipeds of males were significantly longer than those of females (T-test, *p* < 0.05). * indicates a significant difference. (**c**) Volcano plot of DEGs between 4 dpa and 7 dpa stages. (**d**) GO and KEGG enrichment analysis of DEGs between 4 dpa and 7 dpa stages; red boxes represented terms or pathways enriched at 4 dpa, while blue boxes represented those enriched at 7 dpa. (**e**) GSEA results of the 4 dpa and 7 dpa stages. Transcriptome data from males and females were combined for this comparison; only differences among developmental stages were analyzed, *n* = 6.

Following the peeling off of the scab, the regenerative papilla initiates formal regeneration by proliferating and differentiating into a limb bud with indistinct segments. As regeneration progressed, coordinated morphogenesis meticulously sculpted the definitive cheliped structure, followed by segmental expansion and cuticular melanization. Notably, the early development of the papilla was regulated by steroid hormones, which potentially drove the sexual dimorphism observed in regenerated chelipeds by 28 dpa (Figure 3a,b). Male and female transcript profiles were combined for this comparison to exclude sex effects and highlight changes across regeneration stages. Comparative transcriptomic profiling revealed 636 genes differentially expressed between 4 dpa and 7 dpa, with 387 genes up-regulated and 249 genes down-regulated in the 4 dpa group (Figure 3c).

At 4 dpa, the molecular signature focused on structural biosynthesis and robust metabolism. GO analysis showed that DEGs were primarily enriched in chitin-based cuticle development and growth factor activity (Figure 3d). KEGG analysis revealed a high demand for material synthesis, specifically in pyrimidine/purine metabolism and RNA polymerase pathways. Notably, GSEA identified activation of signaling pathways regulating stem cell pluripotency and the foxo signaling pathway (Figure 3e), consistent with the blastema’s role as a convergence of dedifferentiated progenitor cells. By 7 dpa, the landscape shifted toward immune recognition and cytoskeletal remodeling. GO terms were significantly associated with (1 → 3)-β-D-glucan binding and cell surface pattern recognition receptor signaling. At the pathway level, 7 dpa exhibited enrichment in apoptosis and actin cytoskeleton regulation, suggesting active morphological sculpting (Figure 3d). While cAMP and Calcium signaling were enriched at both stages, GSEA specifically highlighted the estradiol pathway at 7 dpa (Figure 3e). This indicates that, following early steroid-regulated initiation, subsequent re-differentiation is further governed by sex hormone signaling.

### 3.4. Sexual Differences in the Transcriptomic Landscape During the Papilla Stage

**Figure 4:**
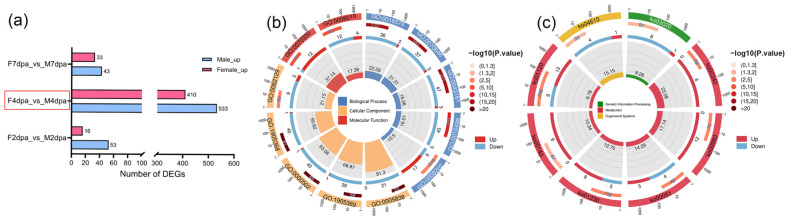
DEGs between sexes during cheliped regeneration in *E. sinensis.* (**a**) Statistics of the number of sex-biased DEGs at each stage, the red box highlights the statistics of differentially expressed genes between sexes at 4 dpa. (**b**) GO enrichment analysis of sex-biased DEGs at the papilla stage. (**c**) KEGG enrichment analysis of sex-biased DEGs at the papilla stage.

To investigate the sexual differences in *E. sinensis* during early limb regeneration, we analyzed the DEGs between sexes across three stages. The results showed a male-biased expression pattern, with more up-regulated genes in males than in females at all stages; the number of DEGs peaked at 4 dpa (Figure 4a). Functional enrichment analysis revealed that male-upregulated genes were significantly enriched in protein modification processes, such as protein deubiquitination (GO:0016579) and protein modification by small protein removal (GO:0070646). In contrast, female-upregulated genes were enriched in aromatase activity (GO:0070330). Regarding cuticle-related terms, males showed enrichment in the structural constituent of chitin-based larval cuticle (GO:0008010) (Figure 4b). Furthermore, KEGG enrichment analysis revealed distinct enrichment patterns between sexes: the complement and coagulation cascades (ko04610) and fructose and mannose metabolism (ko00051) were enriched in males, while steroid hormone biosynthesis (ko00140) and metabolism of xenobiotics by cytochrome P450 (ko00980) were enriched in females (Figure 4c).

### 3.5. Antp and Sex-Differential Key DEGs Revealed by Cluster-Based Screening

**Figure 5:**
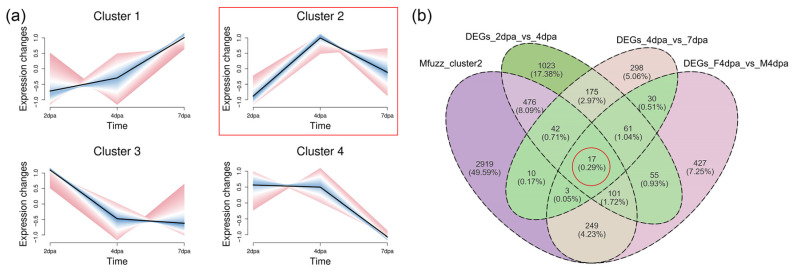
Identification of genes with peak expression at the papilla stage through Mfuzz clustering and DEGs intersection. (**a**) Cluster analysis of genes based on expression patterns. (**b**) Venn Diagram of the intersection between cluster 2 genes, DEGs across different regeneration stages and sex-DEGs at 4 dpa.

All genes identified by transcriptome sequencing that met the screening criteria were categorized into 4 clusters based on their expression patterns (Figure 5a). Given that 4 dpa was a critical transitional phase during the early stage of cheliped regeneration and also a pivotal period for sexual differentiation, we focused on the genes with relatively high expression levels at 4 dpa. Among these clusters, the genes in cluster 2 exhibited the highest expression levels at 4 dpa in terms of their expression patterns, and this module contained 3817 genes. By taking the intersection of DEGs across different regeneration stages, DEGs between sexes at 4 dpa, and genes within Cluster 2, we identified 17 key genes (Figure 5b, Table 2). Among these were *Antp*, *Cyp2L1-like*, *CpAMP1A-like* and *Nedd4-like*.

### 3.6. Validation of the Key DEGs and Their Expression Dynamics During Regeneration

**Figure 6:**
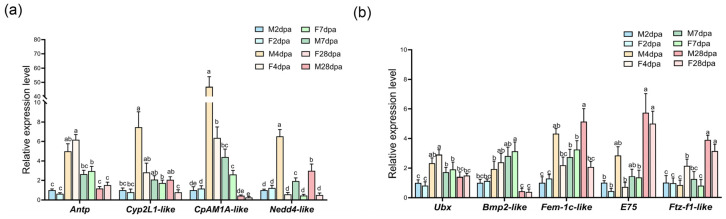
qRT-PCR validation of key DEGs. (**a**) qRT-PCR validation of overlapping DEGs between different regeneration stages and sexes at 4 dpa, including *Antp*, *Cyp2L1-like*, *CpAMP1A-like* and *Nedd4-like*, *n* = 4. (**b**) qRT-PCR validation of other key DEGs. *Ubx* and *Bmp2-like* were DEGs between different stages, *Fem-1c-like*, *Ftz-f1-like*, and *E75* were sex-biased DEGs at 4 dpa, *n* = 4. Statistical analysis was performed using mixed-model ANOVA. Shapiro–Wilk and Levene’s tests were used to assess normality and variance homogeneity, respectively. *Antp*, *CpAMP1A-like*, *Cyp2L1-like*, and *E75* were analyzed with Dunnett T3 test due to unequal variances, while the remaining genes were compared using Tukey’s test. Different lowercase letters above the bars indicate significant differences between groups.

To verify the reliability of the transcriptomic data, several representative genes were selected from the intersection analysis for validation, including *Antp*, *CpAMP1A-like*, *Cyp2L1-like*, and *Nedd4-like*. All these genes reached their peak expression levels at 4 dpa and exhibited significant sex-based differences. Notably, *Nedd4-like* expression was consistently higher in males than in females throughout the cheliped redifferentiation stage (Figure 6a). Furthermore, we validated the expression levels of sex-differential DEGs identified at 4 dpa, such as *Fem-1c-like*, *Ftz-f1-like*, and *E75*, along with genes showing significant differential expression across different regeneration stages, including *Ubx* and *Bmp2-like* (Figure 6b). The qRT-PCR results were highly consistent with the expression trends observed in the RNA-Seq data, confirming the accuracy of our sequencing results. Given the distinct phenotypic differences observed between male and female regenerating claws at 28 dpa, we also performed qPCR analysis on these genes at this later stage to explore their potential roles.

### 3.7. The Key Module and Hub Genes at the Regenerative Papilla Stage

**Figure 7:**
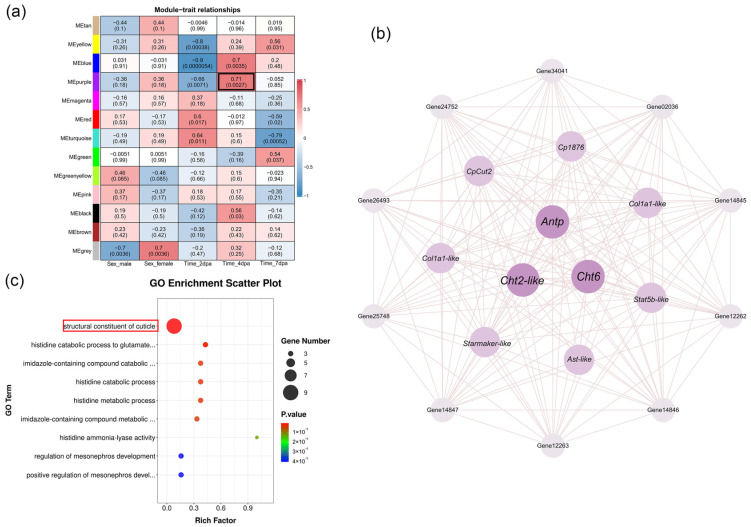
WGCNA and co-expression network of the 4 dpa-associated purple module. (**a**) Heatmap of module-trait relationships. (**b**) Co-expression network of top 20 genes in the purple module. (**c**) Top 20 GO enrichment of the purple module.

WGCNA was performed on transcriptome-derived genes meeting the preset criteria, yielding 12 valid modules. Among these modules, the purple module showed the strongest correlation with 4 dpa (r = 0.71, *p* < 0.05) and comprised a total of 101 genes (Figure 7a). Gene ranking using cytoHubba identified *Antp* as a hub gene with high connectivity within this module. Furthermore, other highly connected genes, such as *Cht6*, *Cht2-like*, and *Cp1876*, were predominantly associated with crustacean cuticle development (Figure 7b). GO enrichment analysis of all genes in the purple module revealed that they were primarily enriched in the functional category of structural constituent of cuticle (Figure 7c).

### 3.8. Effects of Antp RNAi on Cheliped Regeneration in E. sinensis

**Figure 8:**
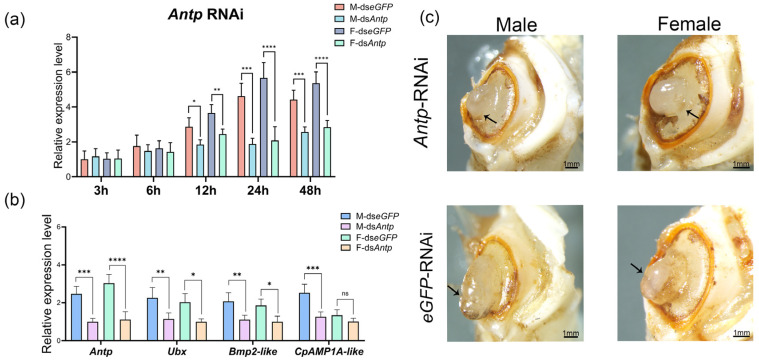
RNAi of *Antp* in *E. sinensis* chelipeds. (**a**) RNAi efficiency assay for *Antp* across time points, showed maximum efficiency at 24 h, *n* = 4. (**b**) Expression changes in target genes after *Antp* RNAi, *n* = 4. (**c**) Morphological phenotypes of regenerated chelipeds following three rounds of *Antp* RNAi. Arrows indicated the regions of obscure segment sexual differentiation in the regenerating chelipeds. Statistical comparisons were conducted by ANOVA with Tukey’s post hoc test. The asterisks *, **, ***, and **** in the figure indicate statistically significant differences at *p* < 0.05, *p* < 0.01, *p* < 0.001, and *p* < 0.0001, respectively. “ns” stands for not significant.

Based on mfuzz analyses and WGCNA, *Antp* was identified as a key gene characterized by both differential expression across regeneration stages and significant sex-based differences. To investigate the functional significance of *Antp*, RNAi was performed via dsRNA injections at the scab stage, achieving peak silencing efficiency at 24 h post-injection. Compared to the control group (dsRNA-*eGFP*), *Antp* expression levels in males and females were significantly reduced to 40.56% and 36.40%, respectively (Figure 8a). The results demonstrated that the silencing of *Antp* led to a significant down-regulation of *Ubx* and *Bmp2-like* in both males and females. Notably, the regulatory effect on *CpAMP1A-like* showed a sex-based difference, with its expression being significantly suppressed only in males. These findings suggest that *Antp* exerted sex-based regulatory control over downstream targets. Specifically, *Antp* appeared to play a more predominant or direct role in the regenerative regulatory network of males compared to females, particularly concerning the modulation of cuticle-related genes such as *CpAMP1A-like* (Figure 8b). Following three rounds of dsRNA-*Antp* treatment, the segmentation boundary between the propodus and carpus of regenerating chelipeds was obscure in several individuals, in sharp contrast to the well-defined segmentation observed in the dsRNA-*eGFP* control group (Figure 8c). Specifically, only 16.7% of males and 13.3% of females in the dsRNA-*Antp* group exhibited distinct segment differentiation, whereas 46.2% of males and 44% of females showed clear segmentation in the control group. These results indicate a significant inhibitory effect of *Antp* knockdown on cheliped segment differentiation (Figure S1).

## 4. Discussion

Cheliped regeneration in *E. sinensis* progresses from wound healing to structure-specific morphogenesis. The wound healing stage (2 dpa) is driven by a defensive-reconstructive network, where MAPK activation likely serves as a dual regulator of stress responses and tissue repair [39]. Concurrently, the upregulation of muscle remodeling genes supports blastema formation, consistent with observations in *Cherax destructor* and *Exopalaemon carinicauda* [14,40]. As regeneration advances to the limb bud stage (7 dpa), the role of apoptosis shifts from cellular clearance to active morphogenetic sculpting, a mechanism essential for segmentation that parallels development in *Drosophila* [41,42]. Ultimately, the definitive sexual dimorphism of the regenerated chelipeds become morphologically distinct by 28 dpa.

The papilla stage (4 dpa) represents a pivotal turning point in the transcriptional landscape, marking the transition from an undifferentiated state to rapid proliferation and epidermal specification. This stage is characterized by a strategic redirection of metabolic energy toward chitin biosynthesis and epidermal morphogenesis. Given that chitin and cuticle proteins are indispensable for morphogenesis in insects [43], a similar conserved mechanism likely operates in *E. sinensis* as a decapod crustacean. The concomitant activation of DNA replication and RNA polymerase pathways signify an exponential surge in cellular proliferation, a fundamental prerequisite for blastema establishment that mirrors the conserved proliferative strategies observed in *Ambystoma mexicanum* [44]. Consequently, we identify 4 dpa as the peak of proliferative transcriptional activity. The regenerative program diverges from a shared wound-healing response to a sex-differential trajectory during this critical window. The distinct transcriptional profiles suggest that the papilla stage serves as the mechanistic foundation where physiological resources are allocated differently between sexes to support dimorphic limb regeneration.

Integrated analysis suggests that *Antp* may act as a central hub gene orchestrating limb patterning. Its temporal expression is low at 2 dpa and peaks at 4 dpa. This pattern implies that the subsequent upregulation potentially drives structure-specific differentiation. Studies in mice (*Mus musculus*) have indicated that Hox gene expression may serve as a mechanism that sustains periosteal stem/progenitor cells in a more primitive tripotent state [45]. RNAi-mediated silencing of *Antp* leads to arrested segmental differentiation and the significant downregulation of *Ubx*, *Bmp2-like*, and *CpAMP1A-like*. These results indicate that *Antp* acts synergistically with *Ubx* to define segmentation [46] and may regulate epidermal development via the TGF-β signaling pathway to further modulate limb size [47]. Furthermore, the observation that *Antp* regulates various epidermal-related genes mirrors conserved mechanisms in *Drosophila* and *Bombyx mori*. *Antp* directly binds to cis-regulatory elements of epidermal protein genes to modulate organ development, and *Antp* knockdown-induced downregulation of cuticle proteins in *E. sinensis* likely follows this conserved mode of action [48]. Collectively, our findings position *Antp* as a master regulator that integrates structural patterning with the expression of epidermal genes.

The pronounced sexual dimorphism in cheliped regeneration is driven by sex-specific regulatory networks recruited from the papilla stage. Male regeneration prioritizes the chitinous exoskeleton pathway (*CpAMP1A-like*) and actively recruits male differentiation factors, specifically *Fem-1c-like* and *Nedd4-like*. The transcriptional profiles align with their established biological functions: Fem-1c plays a crucial role in male sexual differentiation in *E. sinensis*, whereas Nedd4 deficiency leads to male-to-female sex reversal in mice [49,50]. Furthermore, the male-biased fructose and mannose metabolism may be associated with the development of a rigid exoskeleton, a pattern that has also been observed in transcriptomic studies of the silkworm *Bombyx mori* [51]. In contrast, the female program emphasizes systemic signaling, characterized by elevated aromatase activity and *Ftz-f1-like* expression. The enrichment of aromatase and *Ftz-f1-like* in females contrasts sharply with the male-biased expression of *Cyp2L1-like* [52,53]. This distinct pattern suggests that the regenerating tissue actively mediates systemic hormonal signals, mirroring mechanisms observed in *Danio rerio* [54]. Notably, cheliped regeneration in *E. sinensis* is inextricably linked to molting behavior. Given this close association, the nuclear receptors *E75* and *Ftz-f1-like* likely integrate ecdysteroid and sex-steroid signaling [55,56]. Consequently, we propose a hierarchical model wherein sex-biased hormonal signals modulate the expression of *Antp* and other development-related genes, which subsequently influence downstream phenotype-related genes to ensure sexually dimorphic morphogenesis.

## 5. Conclusions

This study provides a comprehensive temporal and sex-differential characterization of early cheliped regeneration in *E. sinensis*. Our findings identify the papilla stage (4 dpa) as a pivotal physiological and transcriptional crossroad that underpins the molecular foundation of sexual dimorphism. This divergence is governed by sex-differential networks: males exhibit preferential expression of *Fem-1c-like*, *Cyp2L1-like*, and *Nedd4-like*, while females are characterized by elevated aromatase activity. Within this regulatory framework, the Hox gene *Antp* serves a critical role in orchestrating appendage patterning and exoskeleton assembly with highly co-expressed cuticle-related genes. Ultimately, we speculate a hierarchical regulatory model in which systemic hormonal signals may integrate *Antp* and other sex-biased regulators to promote the precisely structured regeneration of chelipeds. These findings provide novel insights into the interplay between developmental plasticity and sex-differential development in decapod crustaceans.

## Figures and Tables

**Table 1:** Primer sequences used in the RNAi.

Primer Name	Primer Sequence (5′-3′)	Target
ds*Antp*-F	CCGTCTCCCCTCTACCCAT	RNAi
ds*Antp*-T7-F	GATCACTAATACGACTCACTATAGGGCCGTCTCCCCTCTACCCAT	RNAi
ds*Antp*-R	GGTCGGCGTATCTGAAAGGC	RNAi
ds*Antp*-T7-R	GATCACTAATACGACTCACTATAGGGGGTCGGCGTATCTGAAAGGC	RNAi
ds*eGFP*-F	CAGTGCTTCAGCCGCTACC	RNAi
ds*eGFP*-T7-F	GATCACTAATACGACTCACTATAGGGAGTTCACCTTGATGCCGTTCTT	RNAi
ds*eGFP*-R	AGTTCACCTTGATGCCGTTCTT	RNAi
ds*eGFP*-T7-R	GATCACTAATACGACTCACTATAGGGCAGTGCTTCAGCCGCTACC	RNAi

**Table 2:** The key DEGs of the papilla stage.

Gene_id	F4dpa TPM Mean	M4dpa TPM Mean	Nr Annotation
Gene01240	4.01	24.68	Carbohydrate sulfotransferase 10-like
Gene10240	248.76	22.61	Malignant T-cell-amplified sequence 1-like
Gene11122	14.7	5.59	Homeobox protein Antennapedia
Gene11342	4.65	17.91	Uncharacterized protein LOC127009090
Gene16728	4.70	53.64	Phenoloxidase-activating factor 2-like
Gene16729	5.45	87.09	Phenoloxidase-activating factor 2-like
Gene19010	6.82	63.50	Cytochrome P450 2L1-like
Gene19557	2.29	16.19	Uncharacterized protein LOC126999304
Gene20039	2.66	26.26	Serine/threonine-protein kinase 10-like
Gene26176	3.09	31.26	Carbohydrate sulfotransferase 1-like
Gene26954	12.39	476.31	Uncharacterized protein LOC123505112
Gene27467	1.57	39.81	Chitin biosynthesis protein CHS5-like
Gene30614	18.53	4744.12	Hypothetical protein Pcinc 023297
Gene31973	6.84	655.98	Cuticle protein AMP1A-like
Gene31980	3.52	481.62	Cuticle protein AMP1A-like
Gene33857	9.17	85.60	E3 ubiquitin-protein ligase Nedd-4-like
Gene37332	4.92	969.52	Uncharacterized protein LOC126998486

## Data Availability

The transcriptomic data generated in this study have been deposited in the National Center for Biotechnology Information (NCBI) BioProject database under the accession number PRJNA1415725.

## Supplementary Materials

The following supporting information can be downloaded at: https://www.mdpi.com/article/10.3390/ani16060982/s1. Table S1: Primer sequences used in qRT-PCR; Table S2: Sequencing statistics of the transcriptome data; Figure S1: Regeneration status of the different RNAi groups.

## References

[1] Poss K.D. (2010). Advances in understanding tissue regenerative capacity and mechanisms in animals. Nat. Rev. Genet..

[2] Tanaka E.M., Reddien P.W. (2011). The cellular basis for animal regeneration. Dev. Cell.

[3] Jopling C., Boue S., Izpisua Belmonte J.C. (2011). Dedifferentiation, transdifferentiation and reprogramming: Three routes to regeneration. Nat. Rev. Mol. Cell Biol..

[4] Galliot B., Ghila L. (2010). Cell plasticity in homeostasis and regeneration. Mol. Reprod. Dev..

[5] Mokalled M.H., Poss K.D. (2018). A regeneration toolkit. Dev. Cell.

[6] Almazán A., Çevrim Ç., Musser J.M., Averof M., Paris M. (2022). Crustacean leg regeneration restores complex microanatomy and cell diversity. Sci. Adv..

[7] Che S., Gao J., Zhu H., Du J., Cao L., Zheng Y., Xu G., Liu B. (2025). Integrated application of transcriptomics and metabolomics provides insights into the different body-size growth in Chinese mitten crab (*Eriocheir sinensis*). Int. J. Mol. Sci..

[8] The Bureau of Fisheries and Fishery Administration, Ministry of Agriculture, National Fisheries Technology Extension Center, Chinese Society of Fisheries (2025). China Fisheries Statistical Yearbook 2025.

[9] De Giosa M., Czerniejewski P., Tanski A. (2013). Sexual dimorphism in the relative growth of the claw weight of adult Chinese mitten crab (*Eriocheir sinensis*): A generalized least squares approach. Ital. J. Zool..

[10] Xu J.Q., Wu X.G., Zhang P.C., He J., Fan Y.W., Liu M.M., Cheng Y.X. (2016). Growth, Gonadal Development and Secondary Sexual Characteristics of Pond-reared Male Chinese Mitten Crab (*Eriocheir sinensis*) during the Second Year Culture. China J. Zool..

[11] Juanes F., Smith L.D. (1995). The ecological consequences of limb damage and loss in decapod crustaceans: A review and prospectus. J. Exp. Mar. Biol. Ecol..

[12] Fang P., Sheng S., Li Y., Li Y., Mo R., Mei H., Jiang G., Liu W., Liu H. (2025). Personality traits of the territorial crustacean Chinese mitten crab (*Eriocheir sinensis*): Behavioral adaptations to environmental cues. Animals.

[13] Zhang C., Song X., Zhang Q., Pang Y., Lv J., Tang B., Cheng Y., Yang X. (2018). Changes in bud morphology, growth-related genes and nutritional status during cheliped regeneration in the Chinese mitten crab, *Eriocheir sinensis*. PLoS ONE.

[14] Xing C., Wang M., Chen Z., Li Y., Zhou X., Wang L., Zhong Y., Li W., Shen X., Gao H. (2024). Morphological and molecular changes during limb regeneration of the *Exopalaemon carinicauda*. Animals.

[15] Wang J., Chen X., Hou X., Wang J., Yue W., Huang S., Xu G., Yan J., Lu G., Hofreiter M. (2022). Omics data unveil early molecular response underlying limb regeneration in the Chinese mitten crab, *Eriocheir sinensis*. Sci. Adv..

[16] Liu L., Fu Y., Zhu F., Mu C., Li R., Song W., Shi C., Ye Y., Wang C. (2018). Transcriptomic analysis of *Portunus trituberculatus* reveals a critical role for *WNT4* and WNT signalling in limb regeneration. Gene.

[17] Li J., Lv X., Zhang X., Zhao X., Meng Y., Liu S., Fu S., Sun J. (2024). Notch signaling regulates limb regeneration through *Hes1* and *HeyL* in the Chinese mitten crab. Insect Biochem. Mol. Biol..

[18] Su Y., Mou S., Song Y., Zhang H., Zhang Q. (2025). Genome-wide identification of the *TGF-β* superfamily and their expression in the Chinese mitten crab *Eriocheir sinensis*. Sci. Rep..

[19] Angelini D.R., Kaufman T.C. (2005). Comparative developmental genetics and the evolution of arthropod body plans. Annu. Rev. Genet..

[20] Martin A., Serano J.M., Jarvis E., Bruce H.S., Wang J., Ray S., Barker C.A., O’Connell L.C., Patel N.H. (2016). CRISPR/Cas9 mutagenesis reveals versatile roles of Hox genes in crustacean limb specification and evolution. Curr. Biol..

[21] Devi T.R., Shyamala B.V. (2013). Male- and female-specific variants of doublesex gene products have different roles to play towards regulation of Sex combs reduced expression and sex comb morphogenesis in *Drosophila*. J. Biosci..

[22] Bolger A.M., Lohse M., Usadel B. (2014). Trimmomatic: A flexible trimmer for Illumina sequence data. Bioinformatics.

[23] Cui Z., Liu Y., Yuan J., Zhang X., Ventura T., Ma K.Y., Sun S., Song C., Zhan D., Yang Y. (2021). The Chinese mitten crab genome provides insights into adaptive plasticity and developmental regulation. Nat. Commun..

[24] Kim D., Paggi J.M., Park C., Bennett C., Salzberg S.L. (2019). Graph-based genome alignment and genotyping with HISAT2 and HISAT-genotype. Nat. Biotechnol..

[25] Li H., Handsaker B., Wysoker A., Fennell T., Ruan J., Homer N., Marth G., Abecasis G., Durbin R. (2009). The sequence alignment/map format and SAMtools. Bioinformatics.

[26] Pertea M., Pertea G.M., Antonescu C.M., Chang T.C., Mendell J.T., Salzberg S.L. (2015). StringTie enables improved reconstruction of a transcriptome from RNA-seq reads. Nat. Biotechnol..

[27] Wagner G.P., Kin K., Lynch V.J. (2012). Measurement of mRNA abundance using RNA-seq data: RPKM measure is inconsistent among samples. Theory Biosci..

[28] Love M.I., Huber W., Anders S. (2014). Moderated estimation of fold change and dispersion for RNA-seq data with DESeq2. Genome Biol..

[29] Gómez-Rubio V. (2017). ggplot2-elegant graphics for data analysis. J. Stat. Softw..

[30] Buchfink B., Xie C., Huson D.H. (2015). Fast and sensitive protein alignment using DIAMOND. Nat. Methods.

[31] Altschul S.F., Gish W., Miller W., Myers E.W., Lipman D.J. (1990). Basic local alignment search tool. J. Mol. Biol..

[32] Jiang S., Li H., Zhang L., Mu W., Zhang Y., Chen T., Wu J., Tang H., Zheng S., Liu Y. (2025). BioGDB: A comprehensive database for biological functional enrichment analysis and visualization. Nucleic Acids Res..

[33] Kumar L., Futschik M.E. (2007). Mfuzz: A software package for soft clustering of microarray data. Bioinformation.

[34] Chen H., Boutros P.C. (2011). VennDiagram: A package for the generation of highly-customizable Venn and Euler diagrams in R. BMC Bioinform..

[35] Livak K.J., Schmittgen T.D. (2001). Analysis of relative gene expression data using real-time quantitative PCR and the 2^−∆∆CT^ method. Methods.

[36] George D., Mallery P. (2019). IBM SPSS Statistics 26 Step by Step: A Simple Guide and Reference.

[37] Chen C., Wu Y., Li J., Wang X., Zeng Z., Xu J., Liu Y., Feng J., Chen H., He Y. (2023). TBtools-II: A “one for all, all for one” bioinformatics platform for biological big-data mining. Mol. Plant.

[38] Wang X., Liang S., Yang W., Yu K., Liang F., Zhao B., Zhu X., Zhou C., Mur L.A.J., Roberts J.A. (2024). MetMiner: A user-friendly pipeline for large-scale plant metabolomics data analysis. J. Integr. Plant Biol..

[39] Zhang X., Shen G., Guo Y., Zhang X., Zhao Y., Li W., Wang Q., Zhao Y. (2023). Genome-wide identification and analysis of the *MAPKK* gene family in Chinese mitten crab (*Eriocheir sinensis*) and its response to bacterial challenge. Fish Shellfish Immunol..

[40] West J.M., Humphris D.C., Stephenson D.G. (1995). Characterization of ultrastructural and contractile activation properties of crustacean (*Cherax destructor*) muscle fibres during claw regeneration and moulting. J. Muscle Res. Cell Motil..

[41] Barbaste A., Schott S., Benassayag C., Suzanne M. (2023). Dissecting morphogenetic apoptosis through a genetic screen in *Drosophila*. Life Sci. Alliance.

[42] Monier B., Gettings M., Gay G., Mangeat T., Schott S., Guarner A., Suzanne M. (2015). Apico-basal forces exerted by apoptotic cells drive epithelium folding. Nature.

[43] Muthukrishnan S., Mun S., Noh M.Y., Geisbrecht E.R., Arakane Y. (2020). Insect cuticular chitin contributes to form and function. Curr. Pharm. Des..

[44] Tajer B., Savage A.M., Whited J.L. (2023). The salamander blastema within the broader context of metazoan regeneration. Front. Cell Dev. Biol..

[45] Bradaschia-Correa V., Leclerc K., Josephson A.M., Lee S., Palma L., Litwa H.P., Neibart S.S., Huo J.C., Leucht P. (2019). Hox gene expression determines cell fate of adult periosteal stem/progenitor cells. Sci. Rep..

[46] Abzhanov A., Kaufman T.C. (2000). Crustacean (malacostracan) Hox genes and the evolution of the arthropod trunk. Development.

[47] Vora M., Dietz J., Wing Z., George K., Liu J.K., Rongo C., Savage-Dunn C. (2025). Genome-wide analysis of Smad and Schnurri transcription factors in C. elegans demonstrates widespread interaction and a function in collagen secretion. eLife.

[48] Fang C., Xin Y., Sun T., Monteiro A., Ye Z., Dai F., Lu C., Tong X. (2022). The Hox gene Antennapedia is essential for wing development in insects. Development.

[49] Zhu D., Feng T., Mo N., Han R., Lu W., Cui Z. (2023). *Eriocheir sinensis feminization-1c (Fem-1c)* and its predicted miRNAs involved in sexual development and regulation. Animals.

[50] Windley S.P., Mayere C., McGovern A.E., Harvey N.L., Nef S., Schwarz Q., Kumar S., Wilhelm D. (2022). Loss of *NEDD4* causes complete XY gonadal sex reversal in mice. Cell Death Dis..

[51] Tan D., Hu H., Tong X., Han M., Wu S., Ding X., Dai F., Lu C. (2018). Comparative Analysis of the Integument Transcriptomes between Stick Mutant and Wild-Type Silkworms. Int. J. Mol. Sci..

[52] Di Nardo G., Zhang C., Marcelli A.G., Gilardi G. (2021). Molecular and structural evolution of cytochrome P450 aromatase. Int. J. Mol. Sci..

[53] James M.O., Boyle S.M. (1998). Cytochromes P450 in Crustacea. Comp. Biochem. Physiol. C.

[54] von Hofsten J., Olsson P.E. (2005). Zebrafish sex determination and differentiation: Involvement of *FTZ-F1* genes. Reprod. Biol. Endocrinol..

[55] Benrabaa S.A.M., Chang S.A., Chang E.S., Mykles D.L. (2024). Effects of molting on the expression of ecdysteroid responsive genes in the crustacean molting gland (Y-organ). Gen. Comp. Endocrinol..

[56] Morris L.X., Spradling A.C. (2012). Steroid signaling within *Drosophila* ovarian epithelial cells sex-specifically modulates early germ cell development and meiotic entry. PLoS ONE.

