# Supplementary material for: The Papilla Stage as a Critical Molecular Transition: Antp and Sex-Regulatory Network Orchestrate Cheliped Regeneration in Eriocheir sinensis
Source: Animals (Basel). 2026 Mar 21;16(6):982. doi: 10.3390/ani16060982 (PMC13023517; doi:10.3390/ani16060982)
Supplement: Supplementary file 1 [file animals-16-00982-s001.zip › Table S1.pdf]

Table S1. Primer sequences used in qRT-PCR.

Primer name	Forward primer sequence (5′-3′)	Reverse primer sequence (5′-3′)
β-actin	GCATCCACGAGACCACTTACA	CTCCTGCTTGCTGATCCACATC
Rt-*Antp*	CGACAGACCTACACGCGATA	CCCGTTCTCCACTTTGCTCT
Rt-*Ubx*	GCCAAGGACCAGAATGGCTA	GTCATCCCACAGGTGTTCCA
Rt-*Fem-1c-like*	CTCCCGCAAAGACAAAATAGA	GAAGGACACGAAAGAGCTCT
Rt-*Cyp2L1-like*	TGGCAGATGATCGGCAGCATC	TCTCTTGCTGCAGGTGGAGTCC
Rt-*CpAMP1A-like*	AGGGACGAACGCATTCAAGA	ACAAACTTGAGCTCGACCCG
Rt-*E75*	CCTGAAGAAATGCATCGCCG	ACTGTGTGAAGGATGGCTGG
Rt-*Nedd4-like*	TATCGTTCTCGGTCGAGGGT	TGTCAATGCTCACCACCTCC
Rt-*Bmp2-like*	TTCCGTCGACAACAGGAAGG	GACGAACAGCGTCAGAGACA
Rt-*Ftz-f1-like*	TTGATCATCCGGGAGCTTGT	GCAAGTCAGACCAAGAGTGC
