# Supplementary material for: The Papilla Stage as a Critical Molecular Transition: Antp and Sex-Regulatory Network Orchestrate Cheliped Regeneration in Eriocheir sinensis
Source: Animals (Basel). 2026 Mar 21;16(6):982. doi: 10.3390/ani16060982 (PMC13023517; doi:10.3390/ani16060982)
Supplement: Supplementary file 1 [file animals-16-00982-s001.zip › Table S2.pdf]

Table S2. Sequencing statistics of the transcriptome data.

Samples	Clean reads	Clean bases (GB)	Q30 (%)	GC content (%)	Mapped rate (%)
F2dpa_1	20803775	7.09	96.05	48.67	81.49
F2dpa_2	24604730	8.38	96.01	49.44	76.32
F2dpa_3	24492108	8.34	95.90	50.22	76.88
F4dpa_1	21920600	7.47	96.08	50.41	76.24
F4dpa_2	20042247	6.83	95.72	49.95	75.03
F4dpa_3	21644459	7.37	95.57	50.41	77.36
F7dpa_1	19739961	6.72	95.72	49.37	80.17
F7dpa_2	21015671	7.16	95.73	49.81	74.63
F7dpa_3	20561998	7.00	95.64	49.88	81.43
M2dpa_1	17982078	6.12	95.77	49.54	81.90
M2dpa_2	19976609	6.8	95.79	49.56	76.39
M2dpa_3	22275770	7.59	95.85	49.66	82.05
M4dpa_1	18548135	6.31	95.56	50.02	78.49
M4dpa_2	25272565	8.6	95.86	48.72	80.75
M4dpa_3	19606319	6.68	95.63	49.06	80.16
M7dpa_1	22228544	7.57	95.89	48.64	75.83
M7dpa_2	18477026	6.29	95.47	49.73	79.73
M7dpa_3	20742183	7.06	95.51	50.22	80.24
