# Supplementary figures and images for: The Papilla Stage as a Critical Molecular Transition: Antp and Sex-Regulatory Network Orchestrate Cheliped Regeneration in Eriocheir sinensis
Source: Animals (Basel). 2026 Mar 21;16(6):982. doi: 10.3390/ani16060982 (PMC13023517; doi:10.3390/ani16060982)

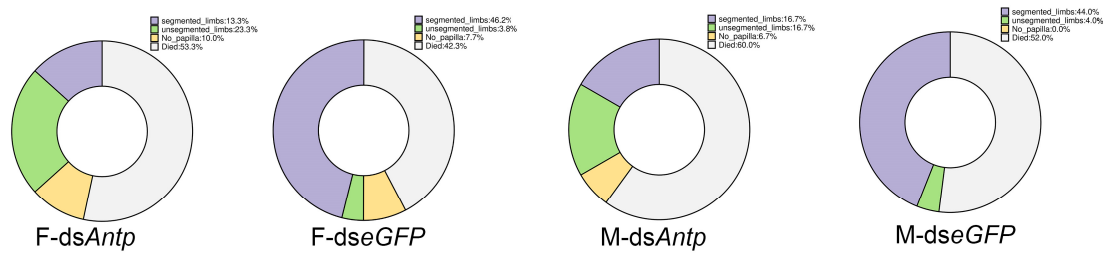

Figure S1. Regeneration status of the different RNAi groups.

Supplement: Supplementary file 1 [file animals-16-00982-s001.zip › Figure S1.pdf]
